# An association between excessive valgus hindfoot alignment and postural stability during single-leg standing in adolescent athletes

**DOI:** 10.1186/s13102-022-00457-7

**Published:** 2022-04-11

**Authors:** Yasunari Ikuta, Tomoyuki Nakasa, Hironori Fujishita, Hiromune Obayashi, Kouki Fukuhara, Tetsuhiko Sakamitsu, Kai Ushio, Nobuo Adachi

**Affiliations:** 1 Department of Orthopaedic Surgery, Graduate School of Biomedical and Health Sciences, Hiroshima University, Kasumi 1-2-3, Minami-ku, Hiroshima, 734-8551 Japan; 2 Sports Medical Center, Hiroshima University Hospital, Hiroshima, Japan; 3 Medical Center for Translational and Clinical Research, Hiroshima University Hospital, Hiroshima, Japan; 4 Department of Rehabilitation Medicine, Hiroshima University Hospital, Hiroshima, Japan

**Keywords:** Adolescent, Athletes, Foot, Postural balance

## Abstract

**Background:**

Diminished balance is associated with the incidence of ankle and lower extremity injuries in adolescents. Although flexible flatfoot is a common foot condition in pediatric and adolescent populations, the association between balance control and foot morphology remain unclear in adolescent athletes.

**Methods:**

Rearfoot angle in the double-limb standing position, body mass index (BMI), and isometric muscle strength related to the knee joint were retrospectively reviewed in 101 adolescent athletes (75 boys and 26 girls) with a mean age of 14.0 years (range 12–17). Postural stability during single-leg standing on static and dynamic platforms was investigated using Balance System SD in 119 feet without functional ankle instability. The participants were divided according to their rearfoot angle into control (less than 7°) and valgus (greater than or equal to 7°) groups. The measured parameters were compared between the control and valgus groups using Welch’s t-test, and P values < 0.05 were considered statistically significant. Multiple regression analysis was conducted to identify the factors that significantly influenced postural control.

**Results:**

The average rearfoot angle was 4.6° in all participants. An excessive valgus rearfoot angle was detected in 53 feet (26.2%). No significant difference was found between the groups in terms of BMI and isometric knee muscle strength. Although no statistical differences were observed in postural stability on the static platform between the control and valgus groups, the valgus group demonstrated poorer postural stability for single-leg standing on the dynamic platform. Multiple regression analysis revealed that BMI and rearfoot angle were significantly associated with a poor postural control on the dynamic platform.

**Conclusions:**

Our findings suggest that excessive rearfoot valgus specifically contributes to the deterioration of postural stability in adolescent athletes, and that rearfoot alignment should be evaluated for the adolescent population to prevent sports-related lower extremity injury.

## Background

Flexible flatfoot is a common foot condition in the pediatric and adolescent populations [1, 2], and the prevalence of pathological flatfoot that required therapeutic attention was reportedly 10.3% in children aged 7–14 years [3]. Pronated foot function affects not only the biomechanics of the foot, but also the entire lower-limb kinematic chain during gait such as peak knee internal rotation and pelvic range of motion [4]. These kinematic alterations can increase the risk of developing musculoskeletal disorders [4]. A systematic review described that flatfoot and high arch were associated with lower extremity injuries compared to the neutral foot type [5], and more frequent ankle sprain was observed in the low arch group than in the normal arch group [6].

Diminished balance is also associated with ankle and lower extremity injuries in high school basketball athletes [7, 8] and female soccer athletes [9]. A significant association between ankle sprains and a positive single-leg balance test was demonstrated prospectively in high school and college age athletes [10]. Several reports have described postural stability in adolescent athletes. Postural stability improved with age from 13 to 18 years, whereas no sex-based differences were found among youth athletes [11]. When dynamic postural stability was investigated using the star excursion balance test in young adolescent secondary school athletes, no significant difference was observed for sex or limb dominance [12]. Athletes between the ages of 10 and 12 years performed worse in the single-leg stance test and demonstrated more asymmetry on the Y-Balance test compared to older athletes, including the 13–15 years and 16–18 years age groups; [13] however, foot morphology was not considered in these studies.

Although both foot morphology and postural stability are associated with lower extremity injury in adolescents, the impact of foot morphology on postural stability is not fully understood in adolescent athletes. The highest incidence of physical injuries has been reported among children aged 10–14 years for both violence-related as well as unintentional sports and recreational injuries [14]. Accordingly, the relationship between foot morphology and postural stability needs to be clarified in pediatric and adolescent populations. Several foot measurement parameters have been reported for clinical assessments. Regression analysis demonstrated that rearfoot angle, footprint index, and truncated arch index were significant predictors of the clinically defined foot type, and the rearfoot angle accounted for 78% of the variance in foot type assessment [15]. Therefore, this study aimed to investigate the association between foot morphology and postural stability, focusing on the rearfoot angle in adolescent athletes. We hypothesized that rearfoot angle is closely associated with postural control in adolescent athletes.

## Methods

This study was approved by the ethics committee of Hiroshima University. The study was conducted retrospectively in adolescent athletes who underwent a medical checkup at Sports Medical Center of Hiroshima University Hospital in 2019. The participants were selected by the Hiroshima City Sports Association, as part of a specially designed program for certified athletes. Informed consent was obtained from the guardians of all participants. This analysis included data from 202 feet in 101 adolescent athletes (75 boys and 26 girls) with a mean age of 14.0 years (range 12–17). They engaged in different sports at a competitive level (Table 1).

**Table 1:** Distribution of sport participation in 101 adolescent athletes

Archery	3	Japanese fencing	8	Soft tennis	8
Basketball	6	Judo	4	Table tennis	6
Badminton	4	Rugby	14	Tennis	4
Handball	7	Sailing	4	Track and field	4
Hockey	9	Skating	4	Water Polo	4
Ice Hockey	4	Soccer	4	Wrestling	4

Number of participants. Adolescent athletes engaged in these sports at a competitive level in their prefecture

Sports activity levels were evaluated using the ankle activity score (AAS) [16]. The AAS consisted of 53 sports, three working activities, and four general activities. Scores of 0–10 points were assigned depending on the type and level of physical activity, with 0 and 10 points indicating the lowest and highest activity level, respectively [16]. The leg used to shoot a ball on a target was defined as the self-reported dominant leg [17]. The functional ankle instability was assessed using the Cumberland ankle instability tool (CAIT) [18]. Participants with a score of < 26 were defined as having functional ankle instability [19]. No major foot deformity, such as rigid flatfoot, clubfoot, or neuromuscular disease was identified in these participants. Participants with a history of concussion were not included in this study.

### Foot alignment and morphology

**Fig. 1:**
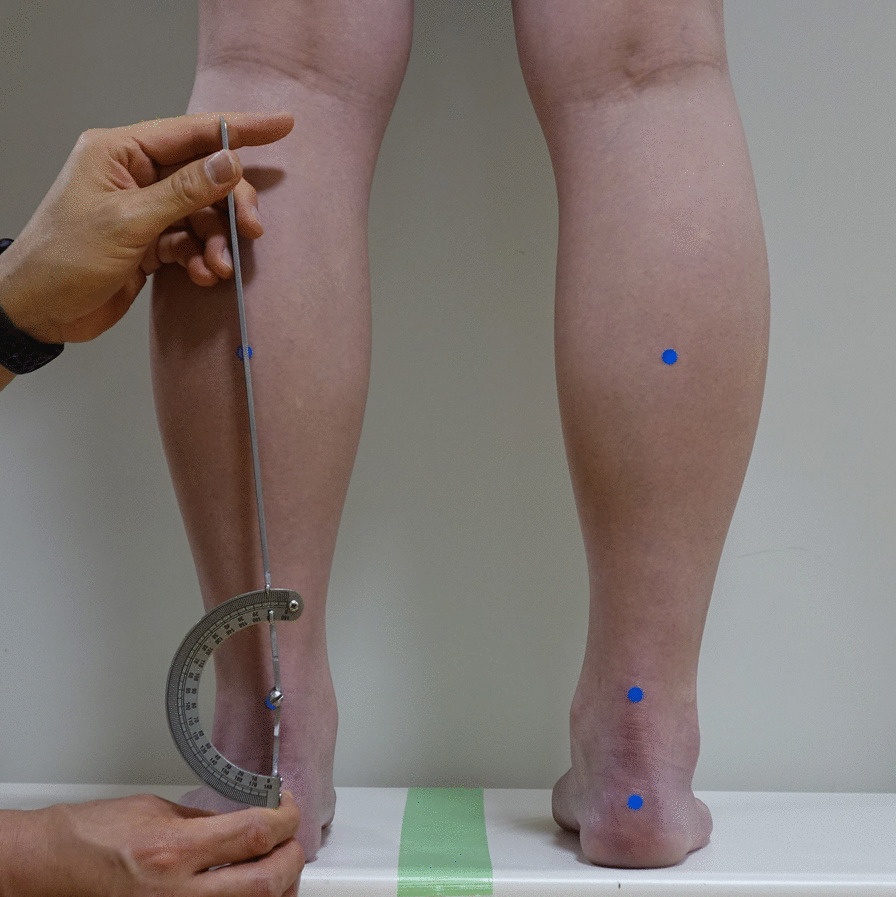
The rearfoot angle was measured using a two-arm goniometer in the double-limb standing position with full weight bearing. Dot stickers were put as references for bisection of the calcaneus and the distal leg

Rearfoot angle was measured using a two-arm goniometer in the double-limb standing position with full weight bearing. The angle was formed by the bisection of the distal one-third of the leg and a longitudinal line that bisected the posterior aspect of the calcaneus [20]. (Fig. 1) A rearfoot angle of more than 7° was defined as excessive valgus heel alignment in the present study [21].

Static foot posture was evaluated by normalized navicular height truncated (NNHT), which was calculated as the navicular height divided by the truncated foot length [22]. Navicular height was measured as the perpendicular distance between the ground and the navicular tuberosity, which was identified as the palpable medial prominence of the midfoot. Truncated foot length was defined as the perpendicular distance from the posterior aspect of the heel to the first metatarsophalangeal joint. The participants maintained a resting stance position with equal weight on both feet during the measurement. A lower NNHT indicates collapse of the medial longitudinal arch.

### Subdivision of participants

Twenty-four participants could not undergo the measurement of postural stability because of equipment malfunction. Consequently, measurements were performed on 77 participants. Thirty-five feet with functional ankle instability were excluded because ankle instability can affect postural stability [23, 24]. In total, 119 feet (bilateral 112 feet, unilateral 7 feet) from 63 participants were included in the subsequent analysis. The participants were divided according to their rearfoot angle into a control group (less than 7°) and valgus group (greater than or equal to 7°) [21].

### Isometric muscle strength

Isometric muscle strength for knee extension and flexion was measured using the Biodex System 4 Dynamometer (Biodex Medical Systems, New York, USA) at an angular velocity of 60°·s. Participants were seated in the dynamometer according to the manufacturer’s guidelines, and five repetitions were conducted after a trial practice. A rest time of more than 30 s was ensured between each measurement. Among the measured values for each repetition, the highest ratio of peak torque to body weight was used in the subsequent analysis. This measurement was conducted for each participant after the postural stability task.

### Postural stability analysis

Balance control ability was assessed for each lower limb using the Balance System SD (Biodex Medical Systems, New York, USA) [25–27]. This system is composed of a platform that has a diameter of 55 cm and tilts 20° from the horizontal in all directions. The measurements were performed on a locked static platform and a dynamic platform with the most stable level 12. The participants maintained their position on the center of the platform for 30 s in single-leg standing position with eyes open, bared feet, and knees slightly flexed. They performed the task three times after a trial practice. A rest time of 1 min was ensured between the measurements. This system provides scores that represent postural stability, including the anterior/posterior index (API) in the sagittal plane, medial/lateral index (MLI) in the frontal plane, and overall stability index (OSI). The OSI is considered as an indicator of the overall ability of the participant to balance the platform in all motions during a test [25]. A high score implies greater platform swing that is induced by more body movement and sway due to an unstable posture.

### Statistical analysis

The measured parameters were compared between the control and valgus groups using Welch’s t-test, including rearfoot angle, NNHT, body mass index (BMI), ankle activity score, ratio of peak torque to body weight, and the stability index. *P* values < 0.05 were considered statistically significant. The rearfoot angle was also compared between the dominant and non-dominant legs.

Multiple regression analysis with a stepwise method was performed to identify the factors that significantly influenced postural control. The independent variables included age, sex, leg dominance, rearfoot angle, NNHT, BMI, AAS, isometric muscle strength for knee extension, and knee flexion. Multicollinearity was determined based on the tolerance and variance inflation factor (VIF). Multicollinearity was identified when the VIF value was greater than 10. The R-squared value was employed to measure the goodness of fit of the linear regression model. These analyses were performed using IBM SPSS Statistics for Windows, version 27.0 (IBM Corporation, Armonk, NY, USA).

## Results

### Foot alignment

The average rearfoot angle was 4.6° (95% confidence interval [CI] 4.1, 5.1) in 202 feet, representing all participants. The rearfoot angle showed neutral or valgus alignment (equal to or greater than 0°) in 197 feet, while rearfoot varus (less than 0°) was identified in five feet. An excessive valgus rearfoot angle (≥ 7°) was detected in 53 feet (26.2%). According to the self-reported leg dominance, the average rearfoot angle was 4.2° (95% CI 3.4, 4.9) in the dominant leg and 5.0° (95% CI 4.3, 5.7) in the non-dominant leg. No significant differences were found between the dominant and non-dominant legs.

### Comparison of groups

A greater rearfoot angle and lower NNHT were observed in the valgus group than in the control group, whereas no significant differences were found in the age and BMI between the two groups. A higher ankle activity score was identified in the valgus group compared to that of the control group (7.5 vs 6.6%, *p* = 0.02). No significant differences were observed in the isometric muscle strength for both knee extension and flexion between the two groups (Table 2).

**Table 2:** Demographic data of the control and valgus groups

	Control group (n = 85)	Valgus group (n = 34)	*P* value
Age (range)	13.8 ± 1.9 (12–17)	14.1 ± 1.3 (12–17)	0.4
RFA	3.0 ± 3.1 (2.3–3.6)	8.3 ± 1.7 (7.7–8.8)	< 0.001
NNHT	0.267 ± 0.028 (0.26–0.272)	0.236 ± 0.037 (0.224–0.249)	< 0.001
BMI	19.9 ± 3.2 (19.2–20.6)	21.1 ± 4.2 (19.6–22.5)	0.17
Ankle activity score	6.6 ± 1.9 (6.2–7.0)	7.5 ± 1.9 (6.9–8.2)	0.02
Muscle strength (Nm/kg)			
Knee extension	219.6 ± 41.6 (210.6–228.6)	220.1 ± 49.3 (202.9–237.2)	0.96
Knee flexion	108.2 ± 30.8 (101.6–114.9)	111.0 ± 32.3 (99.7–122.3)	0.66

Statistical analyses of the control and valgus groups were performed using Welch’s t-test. Age is presented as the mean (range). RFA, NNHT, BMI, ankle activity score, and muscle strength are presented as means ± standard deviation and 95% confidence intervals

RFA, rearfoot angle; NNHT, normalized navicular height truncated; BMI, body mass index

Regarding postural stability, no significant differences were found in the API, MLI, and OSI on the static platform between the control and valgus groups. In contrast, the API, MLI, and OSI were significantly higher in the valgus group than in the control group on the dynamic platform. Thus, the valgus group demonstrated lower postural stability in single-leg standing on the dynamic platform (Table 3).

**Table 3:** Postural stability during single-leg standing on the static and dynamic platform

	Control group	Valgus group	*P* value
Static platform			
OSI	0.98 ± 0.54 (0.86–1.09)	0.99 ± 0.3 (0.89–1.1)	0.85
API	0.69 ± 0.45 (0.59–0.78)	0.7 ± 0.21 (0.62–0.77)	0.87
MLI	0.56 ± 0.25 (0.51–0.62)	0.6 ± 0.24 (0.51–0.68)	0.5
Dynamic platform			
OSI	0.69 ± 0.31 (0.63–0.76)	0.87 ± 0.47 (0.71–1.04)	0.04
API	0.54 ± 0.29 (0.48–0.61)	0.74 ± 0.43 (0.59–0.89)	0.02
MLI	0.37 ± 0.22 (0.32–0.42)	0.49 ± 0.3 (0.38–0.59)	0.04

Statistical analyses of the control and valgus groups were performed using Welch’s t-test. OSI, API, and MLI are presented as means ± standard deviation and 95% confidence intervals

OSI, overall stability index; API, anterior–posterior index; MLI, mediolateral index

Multiple regression analysis revealed that high BMI and greater rearfoot angle were significantly associated with a poor postural control on the dynamic platform. No multicollinearity was present among the independent variables. OSI on the dynamic platform (Y) were positively correlated with BMI (X1) and rearfoot angle (X2), and these results yielded the following equation: y = –0.375 + 0.052 X1 + 0.016 X2. The R-squared value was 0.3.

## Discussion

In this study, we identified poor postural stability in adolescent athletes with hindfoot valgus during single-leg standing on a dynamic platform. The rearfoot angle was used to evaluate foot alignment in the present study. Among the foot measurement parameters, rearfoot angle was the best parameter (78% of the variance) for predicting the clinical assessment of foot type [15]. While the rearfoot angle declines to approximately 4° of valgus from 3 to 6 years of age, it remains stable from 6 to 16 years of age, [21, 28, 29] and no sex differences have been observed [21, 30]. Waseda et al. [31] reported medial longitudinal arch development in 10,155 children aged 6 to 18 years. The arch height ratio (navicular height × 100/foot length) was almost flat until 11 years of age, and increased gradually till the age of 18 years in boys. Similarly, the arch height ratio increased from 10 years of age and reached a plateau at 17 years in girls [31]. Taken together, the rearfoot angle that reaches a plateau at a relatively younger age should be considered an appropriate parameter to assess foot alignment in adolescent athletes with a mean age of 14 years, whose foot arch is progressively developing. A previous study demonstrated that the weight-bearing rearfoot angle was valgus within a range of 0°–7° in 95% of 150 children between the ages of 6 and 16 years [21]. In the present study, valgus over 7° of rearfoot angle was identified in 26.2% (53 of 202 feet) of the adolescent athletes. Furthermore, a higher ankle activity score was observed in the valgus group than in the control group. Excessive foot pronation is induced by adaptation to the forces placed on the musculoskeletal system by kinetic and kinematic events during gait or another action [32]. This raises the possibility that regular sports activities lead to excessive rearfoot valgus via repetitive mechanical loading of the ankle in adolescent athletes during growth periods.

Numerous reports have described the relationship between foot morphology and postural stability in young adults [33–37]. Poorer postural control was noted during a single limb stance in participants with pronated feet than in those with neutral feet, and this foot type was defined by the medial longitudinal arch angle and rearfoot angle [36]. Dynamic balance ability was evaluated in healthy university students using a star excursion test, and no significant differences were found in the foot morphology that was classified by the navicular drop test [33]. The relationship between foot deformity and postural stability were investigated using a 2D podoscan and a dynamometer platform in primary school children. The Clarke’s angle, Wejsflog index, and foot width were identified as the essential predictors of postural stability [38]. Although previous reports investigated postural stability and foot morphology in adults and school children, this is the first study to clarify the relationship between postural stability and hindfoot alignment in adolescent athletes. No significant differences were observed during single-leg standing on the static platform between the control and valgus groups. Sports participation affects postural balance and athletes generally have a superior balance ability compared to controls [39]. This might be the reason for the good postural control demonstrated by the study participants on the static platform. However, the valgus group demonstrated poorer postural stability during single-leg standing on the dynamic platform. Joint hypermobility is common in childhood and resolves with increasing age in normal children [40]. Foot pronation is induced by weight loading, and excessive flexibility of the subtalar joint may lead to an unstable support base and ultimately decrease stability of the foot [34]. Therefore, subtalar joint instability may explain the poorer postural stability in the rearfoot valgus group. Additionally, joint proprioception may be attributed to poorer balance control in adolescent athletes with excessive hindfoot valgus. Joint proprioception and kinesthetic awareness could be decreased in individuals with flatfoot due to excessive stress applied to the muscle spindles and tendon sensory mechanisms [35].

Postural balance during single-leg stance was correlated with the ankle range of motion and muscle strength in young athletes [41]. Moreover, childhood obesity imposes a constraint on voluntary aiming movement due to the effects of body weight on postural control [42], and balance control tends to decrease as the BMI increases in children [43]. In the current study, no significant differences were found in isometric knee muscle strength and BMI between the control and valgus groups. Meanwhile, high BMI and greater rearfoot angle were significantly associated with a poor postural control on the dynamic platform. Therefore, our findings suggest that excessive rearfoot valgus specifically contribute to the deterioration of postural stability during single-leg stance on the dynamic platform in adolescent athletes, even though athletes exhibit better balance control compared to non-athletes.

This study has several limitations. First, rearfoot alignment was not evaluated radiographically. Goniometric clinical measurements were employed to assess rearfoot alignment; however, radiographic measurements are generally available to evaluate rearfoot alignment, such as hindfoot alignment view and long axial view [44]. Radiographic measurements are more reliable than goniometric assessments; [45] thus, further investigations, including radiographic assessment, are warranted to validate our current findings. Second, postural stability was evaluated only during single-leg standing. Balance can be subdivided into four types: static/dynamic steady-state, proactive, and reactive balance [46]. The relationship between rearfoot alignment and postural stability should be investigated during sport-specific tasks such as jumping, landing, and cutting in adolescent athletes.

## Conclusions

Excessive valgus hindfoot alignment, greater than or equal to 7°, was identified in 26.2% of adolescent athletes who demonstrated high ankle activity scores. Although no significant differences were observed in the BMI and isometric muscle strength related to the knee joint between the control and valgus groups, poorer postural stability was observed during single-leg standing on the dynamic platform in adolescent athletes with hindfoot valgus. These findings suggest that excessive rearfoot valgus contributes to the deterioration of postural stability in adolescent athletes and that rearfoot alignment should be evaluated for the adolescent population to prevent sports-related lower extremity injury.

## Data Availability

The datasets used and analyzed during the current study are available from the corresponding author on reasonable request.

## Abbreviations

CAIT: Cumberland ankle instability tool
NNHT: Normalized navicular height truncated
OSI: Overall stability index
BMI: Body mass index

## References

[1] Dars S, Uden H, Banwell HA, Kumar S (2018). The effectiveness of non-surgical intervention (Foot Orthoses) for paediatric flexible pes planus: a systematic review: update. PLoS ONE.

[2] Mosca VS (2010). Flexible flatfoot in children and adolescents. J Child Orthop.

[3] Sadeghi-Demneh E, Melvin JMA, Mickle K (2018). Prevalence of pathological flatfoot in school-age children. Foot (Edinb).

[4] Dodelin D, Tourny C, L'Hermette M (2020). The biomechanical effects of pronated foot function on gait. An experimental study. Scand J Med Sci Sports.

[5] Tong JW, Kong PW (2013). Association between foot type and lower extremity injuries: systematic literature review with meta-analysis. J Orthop Sports Phys Ther.

[6] Mei-Dan O, Kahn G, Zeev A (2005). The medial longitudinal arch as a possible risk factor for ankle sprains: a prospective study in 83 female infantry recruits. Foot Ankle Int.

[7] McGuine TA, Greene JJ, Best T, Leverson G (2000). Balance as a predictor of ankle injuries in high school basketball players. Clin J Sport Med.

[8] Plisky PJ, Rauh MJ, Kaminski TW, Underwood FB (2006). Star Excursion Balance Test as a predictor of lower extremity injury in high school basketball players. J Orthop Sports Phys Ther.

[9] Söderman K, Alfredson H, Pietilä T, Werner S (2001). Risk factors for leg injuries in female soccer players: a prospective investigation during one out-door season. Knee Surg Sports Traumatol Arthrosc.

[10] Trojian TH, McKeag DB (2006). Single leg balance test to identify risk of ankle sprains. Br J Sports Med.

[11] Paniccia M, Wilson KE, Hunt A (2018). Postural stability in healthy child and youth athletes: the effect of age, sex, and concussion-related factors on performance. Sports Health.

[12] Holden S, Boreham C, Doherty C, Wang D, Delahunt E (2014). Dynamic postural stability in young adolescent male and female athletes. Pediatr Phys Ther.

[13] Breen EO, Howell DR, Stracciolini A, Dawkins C, Meehan WP (2016). Examination of age-related differences on clinical tests of postural stability. Sports Health.

[14] Conn JM, Annest JL, Bossarte RM, Gilchrist J (2006). Non-fatal sports and recreational violent injuries among children and teenagers, United States, 2001–2003. J Sci Med Sport.

[15] Chuckpaiwong B, Nunley JA, Queen RM (2009). Correlation between static foot type measurements and clinical assessments. Foot Ankle Int.

[16] Halasi T, Kynsburg A, Tállay A, Berkes I (2004). Development of a new activity score for the evaluation of ankle instability. Am J Sports Med.

[17] van Melick N, Meddeler BM, Hoogeboom TJ, Nijhuis-van der Sanden MWG, van Cingel REH (2017). How to determine leg dominance: the agreement between self-reported and observed performance in healthy adults. PLoS ONE.

[18] Hiller CE, Refshauge KM, Bundy AC, Herbert RD, Kilbreath SL (2006). The Cumberland ankle instability tool: a report of validity and reliability testing. Arch Phys Med Rehabil.

[19] Kunugi S, Masunari A, Noh B, Mori T, Yoshida N, Miyakawa S (2017). Cross-cultural adaptation, reliability, and validity of the Japanese version of the Cumberland ankle instability tool. Disabil Rehabil.

[20] Jonson SR, Gross MT (1997). Intraexaminer reliability, interexaminer reliability, and mean values for nine lower extremity skeletal measures in healthy naval midshipmen. J Orthop Sports Phys Ther.

[21] Sobel E, Levitz S, Caselli M, Brentnall Z, Tran MQ (1999). Natural history of the rearfoot angle: preliminary values in 150 children. Foot Ankle Int.

[22] Menz HB, Munteanu SE (2005). Validity of 3 clinical techniques for the measurement of static foot posture in older people. J Orthop Sports Phys Ther.

[23] Docherty CL, Valovich McLeod TC, Shultz SJ (2006). Postural control deficits in participants with functional ankle instability as measured by the balance error scoring system. Clin J Sport Med.

[24] Chen H, Li HY, Zhang J, Hua YH, Chen SY (2014). Difference in postural control between patients with functional and mechanical ankle instability. Foot Ankle Int.

[25] Testerman C, Vander GR (1999). Evaluation of ankle instability using the Biodex Stability System. Foot Ankle Int.

[26] Pereira HM, de Campos TF, Santos MB, Cardoso JR, Garcia Mde C, Cohen M (2008). Influence of knee position on the postural stability index registered by the Biodex Stability System. Gait Posture.

[27] Rein S, Fabian T, Zwipp H, Mittag-Bonsch M, Weindel S (2010). Influence of age, body mass index and leg dominance on functional ankle stability. Foot Ankle Int.

[28] Pfeiffer M, Kotz R, Ledl T, Hauser G, Sluga M (2006). Prevalence of flat foot in preschool-aged children. Pediatrics.

[29] Uden H, Scharfbillig R, Causby R (2017). The typically developing paediatric foot: how flat should it be? A systematic review. J Foot Ankle Res.

[30] Nguyen AD, Shultz SJ (2007). Sex differences in clinical measures of lower extremity alignment. J Orthop Sports Phys Ther.

[31] Waseda A, Suda Y, Inokuchi S, Nishiwaki Y, Toyama Y (2014). Standard growth of the foot arch in childhood and adolescence—derived from the measurement results of 10,155 children. Foot Ankle Surg.

[32] Horwood AM, Chockalingam N (2017). Defining excessive, over, or hyper-pronation: a quandary. Foot (Edinb).

[33] Hyong IH, Kang JH (2016). Comparison of dynamic balance ability in healthy university students according to foot shape. J Phys Ther Sci.

[34] Kim JA, Lim OB, Yi CH (2015). Difference in static and dynamic stability between flexible flatfeet and neutral feet. Gait Posture.

[35] Tahmasebi R, Karimi MT, Satvati B, Fatoye F (2015). Evaluation of standing stability in individuals with flatfeet. Foot Ankle Spec.

[36] Tsai LC, Yu B, Mercer VS, Gross MT (2006). Comparison of different structural foot types for measures of standing postural control. J Orthop Sports Phys Ther.

[37] Cobb SC, Tis LL, Johnson BF, Higbie EJ (2004). The effect of forefoot varus on postural stability. J Orthop Sports Phys Ther.

[38] Szczepanowska-Wolowiec B, Sztandera P, Kotela I, Zak M (2019). Feet deformities and their close association with postural stability deficits in children aged 10–15 years. BMC Musculoskelet Disord.

[39] Hrysomallis C (2011). Balance ability and athletic performance. Sports Med.

[40] Tofts LJ, Elliott EJ, Munns C, Pacey V, Sillence DO (2009). The differential diagnosis of children with joint hypermobility: a review of the literature. Pediatr Rheumatol Online J.

[41] Trajković N, Kozinc Ž, Smajla D, Šarabon N (2021). Relationship between ankle strength and range of motion and postural stability during single-leg quiet stance in trained athletes. Sci Rep.

[42] Boucher F, Handrigan GA, Mackrous I, Hue O (2015). Childhood obesity affects postural control and aiming performance during an upper limb movement. Gait Posture.

[43] McGraw B, McClenaghan BA, Williams HG, Dickerson J, Ward DS (2000). Gait and postural stability in obese and nonobese prepubertal boys. Arch Phys Med Rehabil.

[44] Reilingh ML, Beimers L, Tuijthof GJ, Stufkens SA, Maas M, van Dijk CN (2010). Measuring hindfoot alignment radiographically: the long axial view is more reliable than the hindfoot alignment view. Skeletal Radiol.

[45] Lamm BM, Mendicino RW, Catanzariti AR, Hillstrom HJ (2005). Static rearfoot alignment: a comparison of clinical and radiographic measures. J Am Podiatr Med Assoc.

[46] Shumway-Cook A, Woollacott MH (2017). Motor control: translating research into clinical practice.

